# Minimally invasive versus open partial nephrectomy for complex renal tumors: insights and limitation

**DOI:** 10.1097/JS9.0000000000001101

**Published:** 2024-01-19

**Authors:** Chuan-Yi Kuo, I-Wen Chen, Kuo-Chuan Hung

**Affiliations:** a Department of Anesthesiology, E-Da Hospital, I-Shou University, Kaohsiung City; b Department of Anesthesiology, Chi Mei Medical Center, Liouying, Tainan City; c Department of Anesthesiology, Chi Mei Medical Center, Tainan City, Taiwan

*Dear Editor*,

We read with interest the recent meta-analysis by Li *et al*.^1^ comparing minimally invasive partial nephrectomy (MIPN) to open partial nephrectomy (OPN) for complex renal tumors. The authors performed a systematic review and meta-analysis comparing MIPN and OPN for treating complex renal tumors^1^. The key findings of this comprehensive analysis were that MIPN was associated with shorter hospital stays, less blood loss, and fewer complications than OPN. There were no significant differences in renal function or oncologic outcomes between the two surgical techniques. A complex anatomy can make minimally invasive resection more challenging. This study highlights the benefits of MIPN over the traditional open approach for the treatment of surgically complex renal masses. Accordingly, these results help support the use of MIPN for complex tumors when surgical expertise allows.

The authors found that MIPN was associated with a significantly shorter length of hospital stay compared to OPN, with a weighted mean difference of −1.84 days^1^. This is an important finding, as reducing hospital stays can optimize healthcare resource utilization. However, there are concerns regarding the validity of this result. First, the presence of publication bias in this outcome raises concerns. The authors failed to clarify whether language-based limitations had been imposed. Considering that the majority of studies were from China and English-speaking nations, potentially significant research in other languages might have been overlooked. A clear statement regarding the presence or absence of such restrictions would enhance the reader’s understanding of the thoroughness of the literature review and the selection methodology.

Second, there was a significant heterogeneity in the length of hospital stay (*I* ^2^=91%). Although the authors conducted subgroup analyses using a surgical approach, significant heterogeneity remained^1^. As heterogeneity may sometimes be difficult to explore, further analysis using prediction intervals can provide additional insight^2^. Prediction intervals are important for assessing the expected range of effects in future clinical settings^2^. Unlike confidence intervals, which address sampling errors, prediction intervals incorporate between-study heterogeneity and thus quantify forecasting uncertainty. A 95% prediction interval indicates the range that would contain the true effect in 95% of identical meta-analyses^2^. To address this knowledge gap, we used the raw data from the original meta-analysis^1^ to conduct a prediction interval with Comprehensive Meta-Analysis (Version 4, Biostat; Englewood, New Jersey, USA). The details are shown in Figure 1. The analysis comparing the length of hospital stay for robotic partial nephrectomy (RPN) versus OPN showed a 95% prediction interval ranging from −4.8 to 1.74 days (Fig. 1A). This wide prediction interval includes the possibility of both shorter and longer hospital stays with RPN than with OPN, highlighting the uncertainty and variability in the data. Accordingly, more research is needed to ascertain the true impact of RPNs on hospital stay length compared to that of OPNs. In comparison, when evaluating laparoscopic partial nephrectomy (LPN) versus OPN, the 95% prediction interval for the length of hospital stay ranged from −4.37 to −0.66 days (Fig. 1B). This finding suggests that LPN is likely to result in a shorter hospital stay than OPN, with a high level of statistical confidence. While the original meta-analysis^1^ found that MIPN is associated with a shorter hospital stay than OPN, further analysis using prediction intervals suggests that this benefit may be greater with LPN than RPN when hospital stay is the primary consideration.

**Figure 1:**
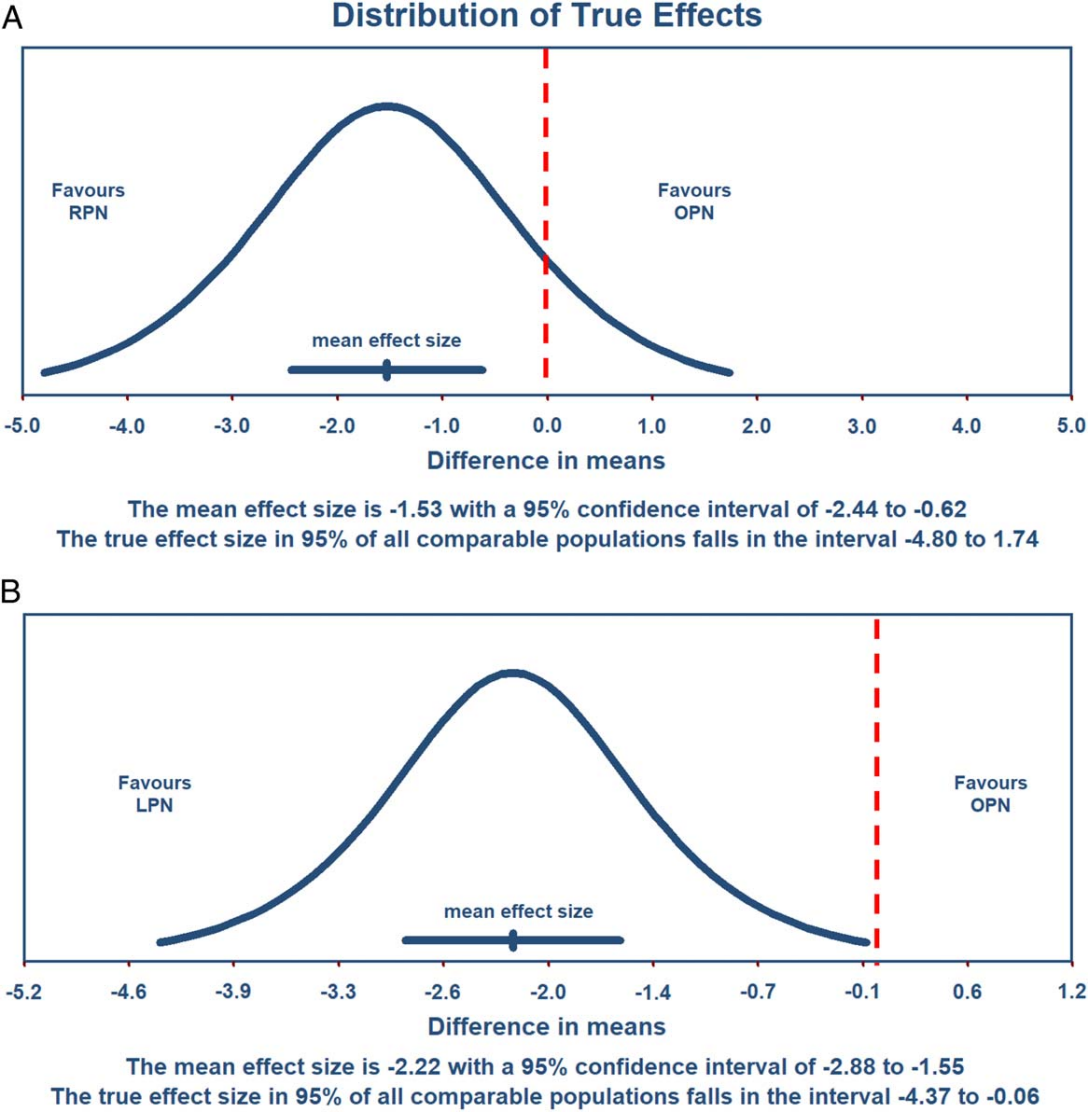
Comparison of hospital stay lengths for different nephrectomy procedures. (A) The graph represents the distribution of true effects compared with robotic partial nephrectomy (RPN) with open partial nephrectomy (OPN). The 95% prediction interval, spanning −4.80 to 1.74 days, encompasses the range within which the true effect size would fall for 95% of comparable populations, suggesting potential variability in outcomes favoring either RPN or OPN. (B) This graph depicts the distribution of the true effects of laparoscopic partial nephrectomy (LPN) versus OPN. The prediction interval of −4.37 to −0.06 days indicates a high probability that LPN will result in a shorter hospital stay than OPN across various populations, with the distribution entirely favoring LPN. Note: Differences in mean values are measured in days. Negative values indicate a shorter hospital stay for minimally invasive procedures (RPN and LPN) than for OPN. The dashed red lines represent the zero-effect threshold.

In conclusion, the meta-analysis by Li *et al*.^1^ provides important insights into the benefits of MIPN over OPN in complex renal tumors. The significant heterogeneity in the length of hospital stays and our prediction interval analysis suggests that the benefits of MIPN, particularly between LPN and RPN, may vary. This nuanced understanding underscores the need for careful surgical technique selection for complex renal tumors and calls for more comprehensive research to better inform clinical decision-making and optimize healthcare resource utilization.

## Ethical approval

Not applicable.

## Consent

Not applicable.

## Sources of funding

No external funding was received for this study.

## Author contribution

C.-Y.K. and K.-C.H.: wrote the main manuscript text; I-W.C.: prepared Figure 1. All authors read and approved the final version of the manuscript.

## Conflicts of interest disclosure

The authors declare no conflicts of interest.

## Research registration unique identifying number (UIN)

Not applicable.

## Guarantor

Kuo-Chuan Hung.

## Data availability statement

The datasets used and/or analyzed in the current study are available from the corresponding author upon reasonable request.

## Provenance and peer review

This paper was not invited.
